# Peroxisome Proliferator-Activated Receptor Gamma Exacerbates Concanavalin A-Induced Liver Injury via Suppressing the Translocation of NF-**κ**B into the Nucleus

**DOI:** 10.1155/2012/940384

**Published:** 2012-12-03

**Authors:** Yuji Ogawa, Masato Yoneda, Wataru Tomeno, Kento Imajo, Yoshiyasu Shinohara, Koji Fujita, Wataru Shibata, Hiroyuki Kirikoshi, Satoru Saito, Koichiro Wada, Shin Maeda, Atsushi Nakajima

**Affiliations:** ^1^Department of Gastroenterology, Yokohama City University Graduate School of Medicine, 3-9 Fukuura, Kanazawa-ku, Yokohama 236-0004, Japan; ^2^Department of Pharmacology, Osaka University Graduate School of Dentistry, 1-8 Yamadaoka, Suita 565-0871, Japan

## Abstract

Peroxisome proliferator-activated receptor-**γ** (PPAR**γ**) has been reported to reduce inflammation and attenuate fibrosis in the liver. In this study, we investigated the effects of PPAR**γ** on the liver injury induced by 20 mg/kg Concanavalin A (Con A). The mice were administered one of the three types of PPAR**γ** ligands (pioglitazone, ciglitazone, and troglitazone) for 1 week, and the serum alanine aminotransferase (ALT) levels at 20 h after Con A injection were significantly elevated in the PPAR**γ** ligand-treated mice. Furthermore, the serum ALT levels after Con A injection in the PPAR**γ** hetero-knock-out mice (PPAR**γ** ^+/−^ mice) were lower than those in the wild-type mice (WT mice). Terminal deoxynucleotidyl transferase dUTP nick end labeling (TUNEL) revealed extensive liver damage induced by Con A in the pioglitazone-treated mice. Electrophoresis mobility shift assay (EMSA) revealed that activation of translocation of nuclear factor- (NF-) **κ**B, which is a suppressor of apoptosis, in the nucleus of the hepatocytes was suppressed in the pioglitazone-treated mice after Con A injection. In this study, we showed that PPAR**γ** exacerbated Con A-induced liver injury via suppressing the translocation of NF-**κ**B into the nucleus, thereby inhibiting the suppression of liver cell apoptosis.

## 1. Introduction

PPARs are members of the nuclear receptor superfamily [1]. Three isotypes designated PPAR*α*, PPAR*β*/*δ*, and PPAR*γ* have been described in mammals [2]. The PPARs form heterodimers with the retinoid X receptor (RXR), and the PPAR-RXR heterodimers, when bound to a ligand, change their conformation and bind to the DNA at the PPAR response elements, which results in gene transcription [3, 4]. PPAR*γ* is expressed in adipose tissue, heart, kidney, skeletal muscle, liver and other organs PPAR*γ* ligands improve insulin resistance and inflammation by increasing serum adiponectin levels [5–7]. Thus, thiazolidinediones (TZDs), which are PPAR*γ* ligands, are widely used in the treatment of type 2 diabetes mellitus (DM). 

Liver injury is caused by various factors such as viral infections, autoimmune reactions, and metabolic disorders. Recently, PPAR*γ* agonists have received attention in relation to the treatment of liver diseases. PPAR*γ* has been reported to reduce hepatic inflammation by decreasing the expression of tumor necrosis factor*α* (TNF-*α*) [8], and suppressing the translocation of NF-*κ*B into the nucleus [9]. Furthermore, the PPAR pathway inhibits the fibrogenic actions in hepatic stellate cells and attenuates liver fibrosis *in vivo* [10, 11]. PPAR*γ* agonists have been reported to be useful in mice and humans with NAFLD [12–14], as PPAR*γ* promotes adipocyte differentiation [15], increases triglyceride storage in adipocyte, and reduces delivery of fatty acids to the liver [9]. However the effect on other liver diseases has not yet been investigated.

In this study, PPAR*γ* ligands and PPAR*γ* ^+/−^ mice were used to confirm the effects of PPAR*γ* on the liver injury induced by Con A. Con A induces serious hepatitis in mice by activating T cells and triggering apoptosis [16, 17]. 

## 2. Materials and Methods

### 2.1. Animal Experiments

Eight-week-old male WT BALB/c mice and eight-week-old male PPAR*γ* ^+/−^ mice on a BALB/c background were purchased from CLEA Japan, Inc. and Jackson Laboratory (Bar Harbor, ME, USA), respectively. All the mice were maintained in filter-topped cages on autoclaved normal chow diet containing 22% protein, 6% fat, and 47% carbohydrate. In the Con A-induced hepatitis model, Con A (Sigma Aldrich, St. Louis, MO, USA; 20 mg/kg) was injected intravenously (i.v.) into mice. First, WT mice (*n* = 6 mice) were fed either a control chow or chow supplemented with one of the two types of PPAR*γ* ligands (ciglitazone (100 mg/kg) and troglitazone (150 mg/kg)) *ad libitum* for 1 week and sacrificed at 20 h after the Con A injection. These doses and duration of treatment with the PPAR*γ* agonists were selected based on the efficacy demonstrated in pilot experiments (data not shown). Subsequently, PPAR*γ* ^+/−^ mice were treated with either control chow or pioglitazone-supplemented chow and sacrificed at 20 h after the Con A injection. Finally, to investigate the effect of PPAR*γ* on the activation of NF-*κ*B induced by Con A, control or pioglitazone-supplemented chow was administered to the WT mice and sacrificed at various time points (0.5 h, 1 h, 3 h, 6 h, and 8 h) after the Con A injection, to obtain nuclear protein. The animal protocols were approved by the Yokohama City University Medical School Guidelines for the Care and Use of Laboratory Animals. 

### 2.2. Biochemistry

Serum alanine aminotransferase (ALT) levels were measured by a local laboratory for clinical examinations (SRL Co, Ltd., Tokyo, Japan). 

### 2.3. Liver Histology

Liver specimens were fixed overnight in buffered formaldehyde (10%) and embedded in paraffin. Paraffin sections were prepared at 5 *μ*m thickness and stained with hematoxylin and eosin (H-E).

### 2.4. Assay for Apoptosis

The apoptotic tumor cells were stained using a TUNEL staining kit, according to the manufacturer's instructions (Wako Pure Chemical, Osaka, Japan). In brief, paraffin sections were digested with 20 *μ*g/mL of proteinase K (Takara, Shiga, Japan) for 15 min at room temperature and reacted with terminal deoxynucleotidyl transferase enzyme for 60 min at 37°C. The sections were then incubated with antidigoxigenin conjugate at room temperature for 30 min, followed by incubation with diaminobenzidine solution.

### 2.5. Electrophoretic Mobility Shift Assay (EMSA)

NF-*κ*B binding was determined by EMSA. We collected liver tissue specimens at various time points (0.5, 1, 3, 6, and 8 h) after the Con A injection. Nuclear protein extracts (10 *μ*g) were prepared using Nuclear Extraction kit (BizScience, Osaka, Japan), according to the manufacturer's instructions. The probe oligonucleotide was 22 bp, double-stranded (5′-GCCTGGGAAAGTCCCCTCAACT-3′) and endlabeled with biotin (Sigma Chemical, St. Louis, MO). DNA-protein complexes were resolved at 80 V for 1 h in a taurine-buffered, native 6% polyacrylamide gel (4% for supershift) and blotted onto a positively charged nylon membrane (Sigma Chemical, St. Louis, MO). Transferred DNA was immediately cross-linked to the membrane on an ultraviolet transilluminator equipped with 312 nm bulbs and detected using horseradish peroxidase-conjugated streptavidin (Light-Shift Chemiluminescent EMSA kit), according to the manufacturer's instructions. 

### 2.6. Statistical Analysis

Data are presented as means ± SD. Differences between the two groups were assessed using the unpaired two-tailed Student's *t*-test; *P* values of <0.05 were considered to denote significance. All statistical analyses were performed using Microsoft Excel and the SPSS 16.0 statistical package (SPSS, Chicago, IL).

## 3. Results and Discussion

**Figure 1:**
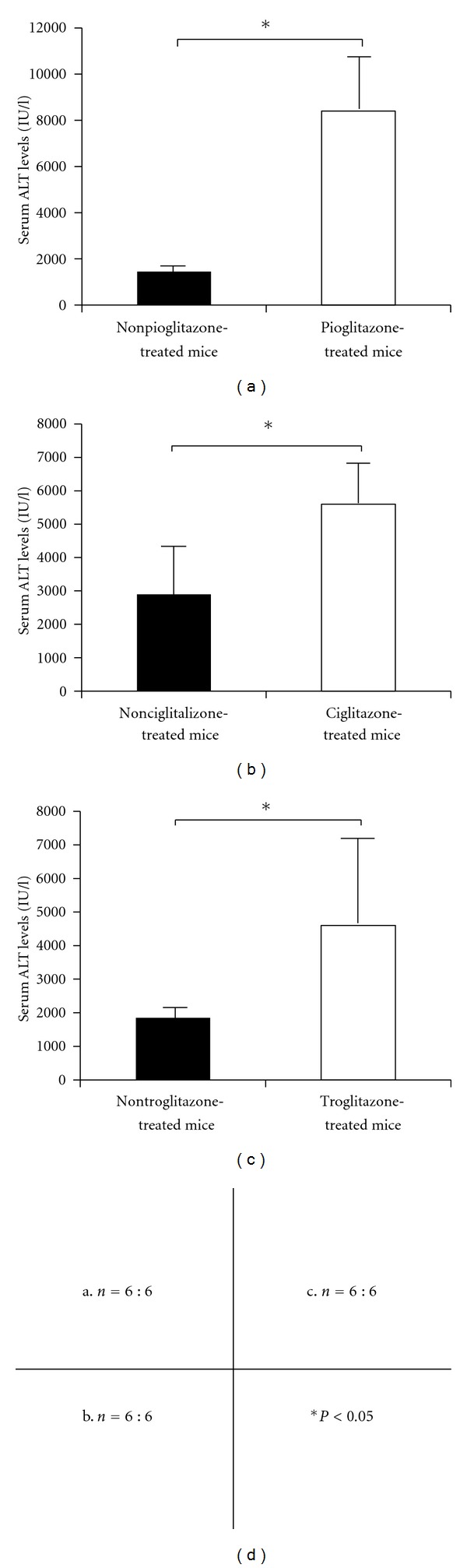
At 20 h after Con A injection, the serum ALT levels in the mice treated with one of the three types of PPAR*γ* ligands (pioglitazone (a), troglitazone (b), ciglitazone (c)) were significantly higher as compared with those in the non-PPAR*γ*-treated mice (*P < 0.05).

**Figure 2:**
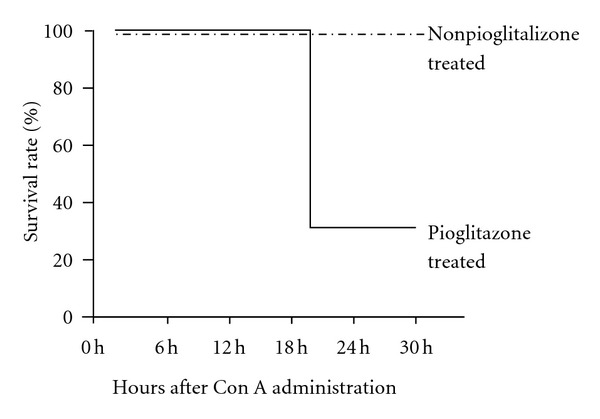
At 20 h after Con A injection, there were no cases of fatality in the nonpioglitazone-treated group of mice, whereas the fatality rate was 70% in the pioglitazone-treated mice.

**Figure 3:**
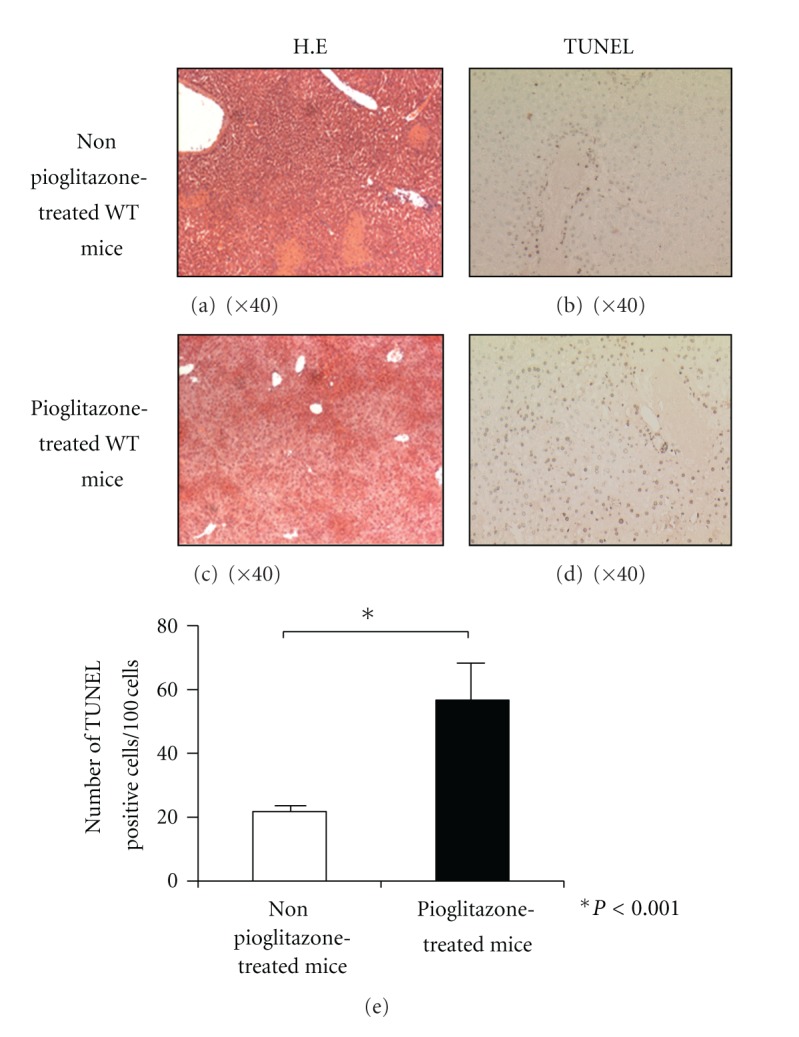
Histopathological examination of liver sections stained with H-E at 20 h after Con A injection revealed more extensive liver necrosis in the pioglitazone-treated mice (c) in comparison with that in the nonpioglitazone-treated mice (a). TUNEL assay at 20 h after Con A injection revealed more extensive liver apoptosis in the pioglitazone-treated mice (d) as compared with that in the nonpioglitazone-treated mice (b). The number of TUNEL-positive cells/100 cells was three-times higher in the livers of the pioglitazone-treated mice as compared with that in the livers of the nonpioglitazone-treated mice (56 ± 8.01 versus 21 ± 1.83, *P* < 0.001).

To assess the degree of liver injury, we analyzed the time course of changes of the serum ALT levels after the Con A injection. Unexpectedly, the serum ALT levels in the pioglitazone- (30 mg/kg) treated mice were significantly higher in comparison with that in the nonpioglitazone-treated mice at 20 h after Con A injection (Figure 1(a)). The survival rate of the nonpioglitazone-treated mice was 100%, while that of the pioglitazone- (30 mg/kg) treated mice was 30% at 20 h after Con A injection (Figure 2). Subsequently, we conducted a histological examination to assess the degree of Con A-induced liver injury at 20 h after Con A injection in the mice treated and not treated with 30 mg/kg of pioglitazone. Histopathological examination of tissue sections stained with H-E revealed that the liver damage was more extensive in the pioglitazone-treated mice as compared with that in the nonpioglitazone-treated mice (Figures 3(a) and 3(c)). To determine the presence and extent of apoptotic cells, we performed TUNEL assay. More TUNEL-positive hepatocytes could be detected in the liver sections of the pioglitazone-treated mice than in those of the control mice (Figures 3(b) and 3(d)). The number of TUNEL positive cells/100 cells in the livers of the pioglitazone-treated mice was three-times higher as compared with that in the livers of the nonpioglitazone-treated mice (56.2 ± 8.0 versus 21.0 ± 1.8, *P* < 0.001). From these results, we hypothesized that PPAR*γ* might actually exacerbate Con A-induced liver injury by intensifying hepatocyte apoptosis. Then, we used two other PPAR*γ* ligands (ciglitazone and troglitazone) to confirm the effect of PPAR*γ*. All of the three PPAR*γ* ligands produced a significant increase of the serum ALT levels in the treated mice as compared with the levels in the untreated mice (Figures 1(a), 1(b), and 1(c)). This result indicates that PPAR*γ* ligands exacerbate Con A-induced liver injury regardless of the kinds. 

**Figure 4:**
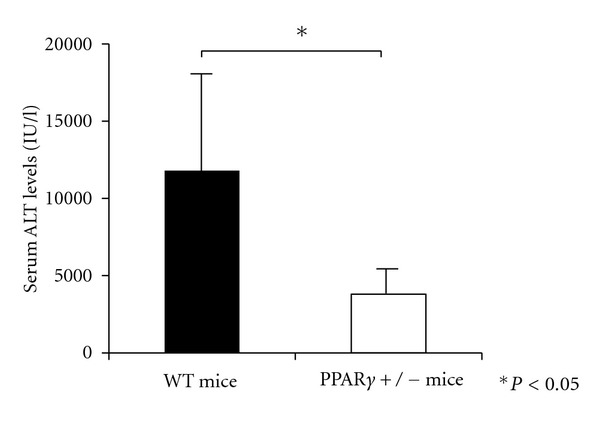
At 20 h after Con A injection, the serum ALT levels in the WT mice were significantly higher than those in the PPAR*γ* ^+/−^ mice.

To evaluate the effect of PPAR*γ* on liver injury, we used PPAR*γ* ^+/−^ mice. The reason for using the heterologous PPAR*γ* ^+/−^ mice was that double knock-out of this gene results in embryonic lethality. Con A-induced liver injury was less extensive in the PPAR*γ* ^+/−^ mice as compared with that in the WT mice (Figure 4). This result suggests the possible involvement of the endogenous PPAR*γ*-mediated pathway in the exacerbation of Con A-induced liver injury.

**Figure 5:**
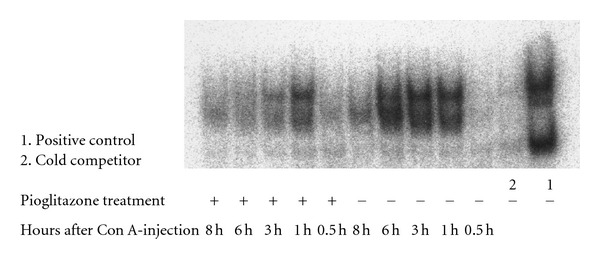
NF-*κ*B binding was determined by EMSA. We collected liver tissue specimens at various time points (0.5, 1, 3, 6, and 8 h) after Con A injection. At 1 h after the Con A injection, NF-*κ*B was activated in the nonpioglitazone-treated mice, whereas the NF-*κ*B activation was suppressed in the pioglitazone-treated mice.

To confirm the apoptosis in the pioglitazone-treated and nonpioglitazone-treated mice after Con A-injection, we analyzed the expression of NF-*κ*B, which is a known suppressor of apoptosis. To determine the quantity of activated NF-*κ*B, we performed EMSA. Activation of NF-*κ*B in the hepatocyte nuclei after Con A-injection was suppressed in the livers of the pioglitazone-treated mice as compared with that in the livers of the nonpioglitazone-treated mice (Figure 5). This result suggests that PPAR*γ* suppresses the translocation of NF-*κ*B into the nucleus, thereby inhibiting the suppression of liver cell apoptosis. Maeda et al. reported that hepatocyte-specific IKK*β* knockout mice exhibit little NF-*κ*B activity and are highly susceptible to liver apoptosis of Con A-induced liver injury [18].

From this study, suppression of PPAR*γ*, such as using PPAR*γ* antagonists may potentially reduce the extent of liver injury.

## 4. Conclusion

In this study, we showed that PPAR*γ* ligands exacerbate Con A-induced liver injury via suppressing the translocation of NF-*κ*B into the nucleus. Con A-induced liver injury in PPAR*γ*-treated mice represents an intensified apoptosis. PPAR*γ* antagonists may be considered as novel candidates for the therapy of liver injury in an intensifying apoptosis model.

## Abbreviations

PPAR*γ*: Peroxisome proliferator-activated receptor-*γ*
NAFLD: Nonalcoholic fatty liver disease
Con A: Concanavalin A
ALT: Alanine aminotransferase
WT mice: Wild-type mice
TUNEL: Terminal deoxinucleotidyl transferase dUTP nick end labeling
EMSA: Electrophoresis mobility shift assay
NF: Nuclear factor
RXR: Retinoid X receptor
TZDs: Thiazolidinediones
DM: Diabetes mellitus
TNF-*α*: Tumor necrosis factor *α*
H-E: Hematoxylin and eosin.
